# Association between P2Y1 and P2Y12 polymorphisms and acute myocardial infarction and ADP-induced platelet aggregation

**DOI:** 10.1186/s12872-023-03075-4

**Published:** 2023-01-21

**Authors:** Chunyan Su, Zhishan Zhang, Jintu Chen, Mengcha Tian, Conglian Wu, Tao Zhang

**Affiliations:** 1 Department of Clinical Laboratory, Quanzhou First Hospital Affiliated to Fujian Medical University, No. 248 East Street, Quanzhou, 362002 Fujian China; 2 Department of Immunology, School of Basic Medical Sciences, Fujian Medical University, 1 Xue Fu North Road, Fuzhou, 350122 Fujian China

**Keywords:** Acute myocardial infarction, P2Y1 gene, P2Y12 gene, Polymorphism, Platelet aggregation

## Abstract

**Background:**

The objective of this study was to investigate the relationship between P2Y1 and P2Y12 genotypes and the risk of acute myocardial infarction (AMI) in the Quanzhou population and to determine associations between P2Y1 and P2Y12 genotypes and ADP-induced platelet aggregation in this population.

**Methods:**

All subjects were screened for P2Y1 (c.1622A > G) and P2Y12 (H1/H2, c.34C > T) polymorphisms by direct DNA sequencing. The maximal platelet aggregation rate (MAR) in AMI patients (n = 61) and healthy control subjects (n = 50) was measured by a PL-12 platelet function analyzer, and adenosine diphosphate (ADP) (5 μmol/L) was used as an agonist.

**Results:**

The haploid H2 allele in the P2Y12 gene was more frequent in patients with AMI than in control subjects (OR 1.887, *P* = 0.005). The P2Y12 H2 haplotype was significantly associated with AMI in the codominant (*P* = 0.008), dominant (OR 2.103, *P* = 0.003), and overdominant models (OR 2.133, *P* = 0.003). After adjusting for potential confounders, H2 haplotype carriers had a 2.132-fold increased risk for AMI (OR 2.132, *P* = 0.012) compared with noncarriers. Moreover, we observed that the ADP-induced MAR in the carriers of the H2 haplotype from the control group was somewhat higher than that in noncarriers of this group (*P* = 0.020). However, we failed to demonstrate that the P2Y1 H1/H2 polymorphism affected ADP-induced MAR in AMI patients. Additionally, P2Y1 c.1622A > and P2Y12 c.34C > T polymorphisms were not associated with the risk of AMI or ADP-induced MAR in either group.

**Conclusions:**

Therefore, our results suggest that the P2Y12 H2 haplotype was associated with a higher risk of AMI, while its effect on increased ADP-induced platelet aggregation remains to be investigated. Thus, the P2Y12 H2 haplotype may be a potential marker for AMI.

## Introduction

Acute myocardial infarction (AMI) is a major cause of morbidity and mortality among cardiovascular diseases (CVDs) in China and is considered a specific type of coronary artery disease (CAD). CAD is a multifactorial disease attributed to the combination of conventional environmental risk factors and genetic factors. Polymorphisms in various genes have been suggested to be involved in the pathogenesis of CAD and influence its susceptibility [1]. Among them, the genetic polymorphisms of P2Y1 and P2Y12, which encode the P2Y1 and P2Y12 receptors, respectively, were found to be associated with thromboembolic events and altered responsiveness to platelet inhibitors [1–4], and they enhance platelet reactivity [5–8]. P2Y1 and P2Y12 are membrane receptors that participate in the process of platelet activation and aggregation via the combination of adenosine diphosphate (ADP), which is considered to be critical for the pathophysiology of atherosclerosis and its complications (e.g., stroke and myocardial infarction).

The P2Y1 gene maps to chromosome 3q21-q25 and consists of a single exon encoding 373 amino acid residues [6, 9]. Several studies confirmed that the c.1622A > G polymorphism of the P2Y1 gene was associated with enhanced platelet reactivity and the antiplatelet drug response [2, 6, 10], but these findings were not confirmed in subsequent studies [7, 11–13]. Moreover, no study has addressed the impact or association of the c.1622A > G polymorphism in the P2Y1 gene and the risk of AMI.

The P2Y12 receptor plays an important role in promoting thrombosis and hemostasis to cerebrovascular events [14]. Deficiency of the P2Y12 receptor impairs coagulation and results in a tendency toward bleeding [15]. The P2Y12 gene is located on chromosome 3q21-q25, and exon 2 encodes the human P2Y12 receptor consisting of 342 amino acid residues. Initially, Fontana et al. first found three introns (i-139C > T, i-744 T > C, i-ins801A) and two exons (c.52G > T and c.34C > T) in the P2Y12 gene [5]. Among these gene polymorphisms, four (i-139C > T, i-744 T > C, i-ins801A, c.52G > T) were shown to be in absolute linkage disequilibrium and were designated haploid H1 (not carrying all four polymorphisms) and haploid H2 (carrying all four polymorphisms) [5]. Several reports have determined that the P2Y12 H2 haplotype is associated with increased platelet aggregation in response to ADP [5, 8] and is associated with a high risk of CAD, ischemic stroke (IS), cerebral infarction and peripheral artery disease[1, 3, 16, 17]. However, these associations were not confirmed in subsequent studies [18, 19]; thus, its role remains unclear.

Few studies have described the relationship between P2Y1 and P2Y12 polymorphisms and AMI. Moreover, the effect of P2Y1 and P2Y12 polymorphisms on the extent of ADP-induced platelet activation in the Quanzhou population in China remains unknown. Therefore, the objective of our study was to investigate the association between the polymorphisms of P2Y1 (c.1622A > G) and P2Y12 (c.34C > T and H1/H2 haplotype) and the risk of AMI and their effect on ADP-induced platelet aggregation.

## Methods

### Study population

Our study recruited patients with AMI (n = 189) from the cardiovascular medicine department of Quanzhou First Hospital in Fujian Province from September 2020 to June 2021. These individuals included 108 males and 81 females (age range from 30 to 83 years). Patients with AMI were diagnosed by medical records, physical examination, electrocardiogram and three-dimensional echocardiography. Moreover, we enrolled age- and sex-matched individuals (n = 194) without a history of cardiovascular and cerebrovascular diseases (e.g., stroke, MI, or CAD) and family history of cardiovascular and cerebrovascular disease as control subjects. The control subjects included 105 males and 89 females (age range from 34 to 81 years). These studies were performed in accordance with the ethical guidelines under protocols approved by the Ethics Committee of Quanzhou First Hospital (Approval Number: [2018]221). Informed consent was obtained from all individual participants included in the study.

### Genotyping

Genomic DNA was extracted using a TIANamp genomic DNA kit (DP304) (TianGen Biotech Co., Beijing, China) according to the manufacturer’s instructions. Subsequently, PCR was performed in a total volume of 25 μl, which included 2 μl DNA template, 1.0 μl each primer (10 µmol/l), 2 μl dNTP mixture, 2.5 μl 10 × ExTaq buffer, 0.1 μl Taq DNA polymerase (5 U/µl) and 16.4 μl distilled water. PCR parameters consisted of initial denaturation at 95 °C for 5 min, followed by 35 cycles at 94 °C for 30 s, 60 °C for 30 s, and 72 °C for 60 s, with a final extension for 7 min at 72 °C. All PCR products were genotyped by direct DNA sequencing on an ABI 3730XL DNA Analyzer (Applied Biosystems, Foster City, CA, USA). The i-744 T > C polymorphism was used as a tag SNP for the H2 haplotype. The primer sets used were as follows: forward primer 5′-GACCACACAGCAGTAGCAGGA-3′ and reverse primer 5′-TGGTCATGAGTTGGCATTCCTCA-3′ for the P2Y12 i-744 T > C polymorphism; forward primer 5′-TCACCTGTTACGACACCACCTCA-3′ and reverse primer 5′-CAACTGTTGAGACTTGCTAGACCTC-3′ for the P2Y1 c.1622A > G polymorphism; and forward primer 5′-TAGAGGAGGCTGTGTCCA-3′ and reverse primer 5′- CTCAGTGGTCCTGTTCCCAG -3′ for the P2Y12 c.34C > T polymorphism.

### Measurement of ADP-induced platelet aggregation

For the aggregation analysis, we collected venous whole blood into tubes containing 3.2% sodium citrate from 61 random AMI patients and 50 healthy volunteers. None of the healthy volunteers smoked, consumed alcohol, took any antiplatelet medicines or suffered from diabetes mellitus or hypertension. Platelet aggregation was measured by a PL-12 platelet function analyzer (SINNOWA Medical Science & Technology Co., Nanjing, China) based on a new platelet count drop method, as described previously [20]. In brief, after the citrated sodium blood sample was placed into the measuring position, the PL-12 counted platelet number prior to and after mixing with inducer ADP (final concentration: 5 μmol/L). PL-12 was counted several times until the lowest level was detected. The maximal platelet aggregation rate (MAR) ratio was calculated using the following formula: MAR = (baseline—lowest platelet count) *100%/baseline.

### Statistical analysis

The results are presented as the mean ± SEM unless indicated otherwise. Quantitative data between two groups were assessed using Student's t test, and qualitative data were assessed with a χ2 test or Fisher’s exact test. The deviation of the Hardy–Weinberg equilibrium for all genotype frequencies in the present study was assessed by χ2 test. The relationship between the variables and AMI was studied using multivariate logistic regression analysis. Statistical analyses were performed using SPSS software version 13.0 (SPSS Inc., Chicago, IL, USA). A value of *P* < 0.05 was considered statistically significant.

## Results

### Characteristics of the participants

Table 1 demonstrates the demographic and clinical characteristics of the AMI patients and control subjects. Our study found that the body mass index (BMI), blood glucose levels, and triglyceride (TG) levels in patients with AMI were significantly higher than those in the control group, while their high-density lipoprotein cholesterol (HDL-C) level was lower than that of the control group (*P* < 0.05). Moreover, other traditional risk factors, such as hypertension and diabetes mellitus, were more prevalent in AMI patients than in control subjects (*P* < 0.05). However, no significant differences (*P* > 0.05) were found between the two groups in terms of age, sex, smoking status, alcohol intake, low-density lipoprotein cholesterol (LDL-C) levels, or total cholesterol (TC) levels.

**Table 1:** Characteristics of AMI and controls in this study

Variable	Controls (n = 194)	AMI (n = 189)	*P* value
Age (years)	58.14 ± 6.78	57.61 ± 10.89	0.569
Men (n, %)	105 (54.1)	108 (57.1)	0.552
BMI (kg/m2)	21.30 ± 1.71	22.94 ± 2.66	0.000*
Hypertension (%)	47 (24.2)	98 (51.9)	0.000*
Diabetes mellitus (n, %)	13 (6.7)	58 (30.7)	0.000*
Blood glucose (mmol/L)	5.57 ± 1.01	6.91 ± 2.51	0.000
TC (mmol/L)	5.08 ± 0.99	5.31 ± 1.49	0.076
TG (mmol/L)	1.58 ± 0.83	1.86 ± 0.99	0.004*
HDL-C (mmol/L)	1.19 ± 0.37	1.09 ± 0.42	0.012*
LDL-C (mmol/L)	3. 52 ± 0.85	3.61 ± 1.08	0.353
Cigarette smoking (n, %)	19 (9.8)	29 (15.3)	0.101
Alcohol intake (n %)	31 (16.0)	40 (22.8)	0.192

*AMI* acute myocardial infarction, *BMI* body mass index, *TC* total cholesterol, *TG* triglycerides, *HDL-C* high-density lipoprotein cholesterol, *LDL-C* low-density lipoprotein cholesterol

*P < 0.05. *P* < 0.05 means a significant difference

### Associations between P2Y1 and P2Y12 polymorphisms and AMI risk

The genotypic distributions of th 3 variants were in accordance with Hardy–Weinberg equilibrium (*P* > 0.05) (Table 2). The distribution of the P2Y1c.1622A > G, P2Y12 c.34C > T and P2Y12 i-744 T > C genotypes and alleles in the control and AMI groups are listed in Table 3.

**Table 2:** The genotype distribution of the Hardy–Weinberg equilibrium in the two groups

SNP	Controls (n = 194)			*P* value	AMI (n = 189)			*P* value
	Genotype (Observed value/Expected value)				Genotype (Observed value/Expected value)			
P2Y1 c.1622A > G	AA (106/101.0)	AG (68/77.9)	GG (20/15.0)	0.0757	AA (102/101.5)	AG (73/74.0)	GG (14/13.5)	0.8507
P2Y12 c.34C > T	CC (124/122.2)	CT (60/63.5)	TT (10/8.2)	0.4421	CC (110/112.0)	CT (71/67.0)	TT (8/10.0)	0.4089
P2Y12 i-744 T > C^#^	TT (163/161.5)	TC (28/31.0)	CC (3/1.5)	0.1750	TT (135/135.4)	TC (50/49.1)	CC (4/1.4)	0.8012

*AMI* acute myocardial infarction, *SNP* single nucleotide polymorphism

^#^i-744 T > C genotype was used to tag the H1/H2 haplotype. TT, TC, and TT genotypes referred to H1/H1, H1/H2, and H2/H2 haplotypes, respectively

No significant association was observed between the P2Y1 c.1622A > G polymorphism and the incidence of AMI in the codominant model (*P* = 0.536), the dominant model (*P* = 0.895), the recessive model (*P* = 0.318), and the overdominant model (*P* = 0.469). Moreover, the frequency of the G allele in AMI patients was 26.7%, which was similar to that in control subjects (G allele occurred in 27.8%, *P* = 0.792) (Table 3).

**Table 3:** Genotypic and allelic distribution of the *P2Y1* and *P2Y12* gene between AMI and contrast groups

SNP	Genetic model	Genotype/allele	Controls (n = 194)	AMI (n = 189)	Odds ratio (95% CI)	*P* value
P2Y1 c.1622A > G	Codominant	AA vs. AG vs. GG	106 (54.6%)/68 (35.1%)/20 (10.3%)	102 (54.0%)/73 (38.6%)/14 (7.4%)	–	0.536
	Allele contrast	A vs. G	280 (72.2%)/108 (27.8%)	277 (73.3%)/101 (26.7%)	0.945 (0.688 ~ 1.299)	0.729
	Dominant	AA vs. AG + GG	106 (54.6%)/88 (45.4%)	102 (54.0%)/87 (45.0%)	1.027 (0.687 ~ 1.536)	0.895
	Recessive	AA + AG vs.GG	174 (89.7%)/20 (10.3%)	175 (92.6%)/14 (7.4%)	0.696 (0.341 ~ 1.422)	0.318
	Over-dominant	AA + GG vs. AG	126 (64.9)/68 (35.1%)	116 (61.4%)/73 (38.6%)	1.166 (0.770 ~ 1.767)	0.469
P2Y12 i-744 T > C^#^	Codominant	TT vs. TC vs. CC	163 (84.0%)/28 (14.4%)/3 (1.5%)	135 (71.4%)/50 (26.5%)/4 (2.1%)	–	0.008*
	Allele contrast	T vs. C	354 (91.2%)/34 (8.8%)	320 (84.7%)/58 (15.3%)	1.887 (1.204 ~ 2.958)	0.005*
	Dominant	TT vs. TC + CC	163 (84.0%)/31 (15.9%)	135 (71.4%)/54 (28.6%)	2.103 (1.279 ~ 3.457)	0.003*
	Recessive	TT + TC vs. CC	191 (98.5%)/3 (1.5%)	180 (97.9%)/4 (2.1%)	1.415 (0.312 ~ 6.409)	0.718
	Over-dominant	TT + CC vs. TC	166 (85.5%)/28 (14.4%)	139 (73.5%)/50 (26.5%)	2.133 (1.275 ~ 3.568)	0.003*
P2Y12 c.34C > T	Codominant	CC vs. CT vs. TT	124 (63.9%)/60 (30.9%)/10 (5.2%)	110 (58.2%)/71 (37.6%)/8 (4.2%)	–	0.383
	Allele contrast	C vs. T	308 (79.4%)/80 (20.6%)	291 (77.0%)/87 (23.1%)	1.151 (0.817 ~ 1.622)	0.442
	Dominant	CC vs. CT + TT	124 (63.9%)/70 (36.1%)	110 (58.2%)/79 (41.8%)	1.272 (0.843 ~ 1.920)	0.251
	Recessive	CC + CT vs. TT	184 (94.8%)/10 (5.2%)	181 (95.8%)/8 (4.2%)	0.813 (0.314 ~ 2.107)	0.670
	Over-dominant	CC + TT vs. CT	134 (69.1%)/60 (30.9%)	118 (62.4%)/71 (37.6%)	1.344 (0.880 ~ 2.052)	0.171

*SNP* single nucleotide polymorphism, *CI* confidence interval, *OR* odds ratio

^#^i-744 T > C genotype was used to tag the H1/H2 haplotype. TT, TC, and TT genotypes referred to H1/H1, H1/H2, and H2/H2 haplotypes, respectively

*P < 0.05. *P* < 0.05 means a significant difference. ^#^ i-744 T > C genotype was used to tag the H1/H2 haplotype. TT, TC, and TT genotypes referred to H1/H1, H1/H2, and H2/H2 haplotypes, respectively

For the P2Y12 gene, the H1/H2 haplotype and c.34C > T polymorphism were selected for analysis in our study. The genotypic frequencies of the P2Y12 H1/H2 haplotype were significantly associated with AMI risk in the codominant model (*P* = 0.005), the dominant model (OR 2.103, 95% CI 1.279–3.457, *P* = 0.003), and the overdominant model (OR 2.133, 95% CI 1.275–3.568, *P* = 0.003). Moreover, the frequencies of the haploid H2 allele were higher in AMI cases than in controls (15.3% vs. 8.8%, OR 1.887, 95% CI 1.204–2.958, *P* = 0.005).

The genotypic distribution of c.34C > T, the other P2Y12 polymorphism, was not found to be associated with the occurrence of AMI in the codominant model (*P* = 0.383), the dominant model (*P* = 0.251), the recessive model (*P* = 0.670), and the overdominant model (*P* = 0.171). Moreover, the frequencies of the C and T alleles in AMI patients were 73.2% and 26.7%, respectively, which were similar to those in control subjects (C allele occurred in 79.4%, T allele occurred in 20.6%, *P* = 0.442).

### Logistic regression analysis of risk factors for AMI

In the multiple regression analysis, we included the prediction of BMI, hypertension, diabetes, TG level, HDL-C level and the P2Y12 H1/H2 genotype. Logistic regression analysis showed that BMI, hypertension, diabetes and haploid H2 allele carriers were important independent regulatory factors for AMI (*P* < 0.05) (Table 4). Individuals who were haploid H2 allele carriers had an ~ 2.054-fold increase in the development of AMI compared with noncarriers after adjustment for other covariates (OR 2.132, 95% CI 1.180–3.854, *P* = 0.012).

**Table 4:** Multivariate logistic regression analysis of the major risk factors for AMI

Risk factor	OR	95%*CI*	*P* value
BMI (kg/m^2^)	1.495	1.314–1.701	0.000*
Hypertension	3.616	2.177–6.007	0.000*
Diabetes mellitus	7.885	3.799–16.367	0.000*
TG	1.376	0.838–2.259	0.207
HDL-C	0.648	0.406–1.035	0.069
P2Y12 H1/H2 genotype	2.132	1.180–3.854	0.012*

*BMI* body mass index, *TG* triglycerides, *HDL-C* high-density lipoprotein cholesterol, *CI*, confidence interval, *OR* odds ratio

*P < 0.05. *P* < 0.05 means a significant difference

### Effect of P2Y1 and P2Y12 polymorphisms on platelet aggression in response to ADP

We found that individuals carrying the haploid H2 allele were more likely to have a higher MAR in response to ADP than noncarriers (48.53 ± 5.35% vs. 44.05 ± 4.99%, *P* = 0.020) (Table 5). In contrast, ADP-induced MAR did not differ significantly between AMI patients with or without the haploid H2 allele (84.21 ± 1.19% vs. 84.02 ± 0.83%, *P* = 0.899) (Table 5). However, we failed to find significant differences in ADP-induced MAR between carriers and noncarriers of the P2Y1 1622G allele as well as the P2Y12 34 T allele (Table 5).

**Table 5:** Effect of *P2Y1* and *P2Y12* genotypes on MAR in response to 5 μmol/L ADP among healthy subjects

SNP	Genotype	Healthy subjects		*P* value	AMI		*P* value
		No	MAR (%)		No	MAR (%)	
P2Y1 c.1622A > G	AA	28	44.85 ± 5.64		38	83.87 ± 0.87	
	AG + GG	22	44.86 ± 4.95	0.995	23	84.43 ± 1.09	0.688
P2Y12 H1/H2	H1/H1	41	44.05 ± 4.99		42	84.02 ± 0.83	
	H1/H2 + H2/H2	9	48.53 ± 5.35	0.020*	19	84.21 ± 1.19	0.899
P2Y12 c.34C > T	CC	32	44.56 ± 5.15		43	83.51 ± 0.86	
	CT + TT	18	45.38 ± 5.65	0.605	18	85.44 ± 0.96	0.281

*SNP* single nucleotide polymorphism

*P < 0.05. *P* < 0.05 means a significant difference

## Discussion

The possible role of genetic variations in the P2Y12 and P2Y1 genes in AMI susceptibility has not yet been thoroughly investigated. Moreover, their effect on ADP-induced platelet aggression remains elusive. In this study, we found that the P2Y12 H2 haplotype was associated with a higher risk of AMI. Additionally, the P2Y12 H2 haplotype displayed markedly increased ADP-induced MAR in healthy adults, but their association was not confirmed in AMI patients.

In our study, the frequency of the 1662G allele was 27.8%, which is consistent (P > 0.05) with the findings in Korea (31%) and in the Hunan Province of China (30.7%) (Table 6) [7, 20]. In the analysis of Yi et al. [21], the P2Y1 c.1622A > G polymorphism was not associated with IS susceptibility. Supported by this finding, our findings indicated that the P2Y1 c.1622A > G polymorphism did not contribute to the risk of AMI. However, we failed to demonstrate that the P2Y1 c.1622A > G polymorphism affected ADP-induced platelet aggregation in both healthy adults and AMI patients. Simon et al. reported that P2Y1 c.1622A > G was associated with increased platelet aggregation in healthy adults from northern Europe [6], but this result was not confirmed in a Korean population [7]. Patients with CAD carrying the 1622 G/G genotype of the P2Y1 gene had significantly higher levels of arachidonic acid-induced platelet aggregation than noncarriers in Caucasian populations, while another study showed that P2Y1 1622A > G did not contribute to clopidogrel resistance in Indian populations [10, 22].

**Table 6:** Distribution of *P2Y1* and *P2Y12* genotypes and alleles among different ethnic groups

SNP		Total (n)	Genotype	Allele	Author
P2Y1 c.1622A > G			AA/AG/GG	A/G	
	Present study	194	106 (54.6%)/68 (35.1%)/20 (10.3%)	280 (72.2%)/108 (27.8%)	Present
	Korea	415	71 (44.9%)/76 (48.1%)/11 (7.0%)*	218 (69.0%)/98 (31.0%)	Kim et al. [7]
	Hunan Province of China	323	155 (48.0%)/138 (42.7%)/30 (9.3%)	448 (69.3%)/198 (30.7%)	Li et al. [20]
P2Y12 i-744 T > C			TT/TC/CC	T/C	
	Present study	194	163 (84.0%)/28 (14.4%)/3 (1.5%)	354 (91.2%)/34 (8.8%)	Present
	South India	221	185 (83.7%)/35 (15.8%)/1 (0.5%)	405 (91.6%)/37 (8.4%)	Priyadharsini et al. [1]
	Western India	102	87 (85.2%)/12 (11.8%)/3 (2.9%)	186 (91.2)/18 (8.8%)	Shalia et al. [21]
	Egypt	40	39 (97.5%)/1 (2.5%)/0 (0%)*	79 (98.8%)/1 (1.2%)*	Zoheir et al. [8]
	Iran	112	1 (0.9%)/8 (7.1%)/103 (92.0%)*	10 (4.5%)/214 (95.5%)*	Azarpira et al. [23]
	Korea	415	283 (68.2%)/125 (30.1%)/7 (1.7%)*	691 (83.3%)/139 (16.7%)*	Kim et al. 2013 [7]
P2Y12 c.34C > T			CC/CT/TT	C/T	
	Present study	194	124 (63.9%)/60 (30.9%)/10 (5.2%)	308 (79.4%)/80 (20.6%)	Present
	Korea	415	83 (52.5%)/66 (41.8%)/9 (5.7%)	232 (73.4%)/84 (26.6%)	Kim et al. 2013 [7]

*P < 0.05. *P* < 0.05 means a significant difference

Our results showed that the frequency of the haploid H2 allele was 8.8% in control subjects. This frequency was similar to that in reports on South Indian (8.4%) and Western Indian (8.8%) populations [1, 21] but different from that in reports on Egyptian, Iranian, and Korean populations (Table 6) [7, 8, 23]. In the present study, the frequency of the haploid H2 allele in the AMI group was significantly higher than that in the control group (*P* = 0.005). Moreover, the P2Y12 H2 haplotype was positively associated with AMI in the codominant, dominant, and overdominant models (*P* < 0.05). The association between the P2Y12 H2 haplotype and AMI susceptibility remained significant after adjustment for multiple comparisons by multivariate logistic regression (OR 2.132, 95% CI 1.180–3.854, *P* = 0.012). Therefore, we conclude that carrying the haploid H2 allele may be one of the risk factors for AMI, which adds very valuable information to this field. Therefore, carrying the H2 haplotype in patients may require closer clinical follow-up. A recent study by Lu et al. [3] showed that carriers with H2 alleles have a higher propensity for cerebral infarction in Sandong, China, and the higher risk of ischemic events in association with the i-744 T > C polymorphism was confirmed in a large meta-analysis summarizing available data up to 2019 [24]. Additionally, several previous studies have demonstrated an association between the P2Y12 H2 haplotype and CA [1, 17]. However, a prospective analysis performed on initially healthy American men failed to find a relationship between the P2Y12 H2 haplotype and the risk of MI, IS, and venous thromboembolism [18]. These conflicting results may be due to allelic heterogeneity, case‒control selection criteria, and different population backgrounds. In addition, the development of cardiovascular events may be determined by multiple genetic factors and environmental factors [25, 26], rather than single-gene polymorphism of P2Y12 [1, 27], with regard to the complexity of atherosclerosis etiology. Therefore, it may be crucial to analyze the effects of gene‒gene interactions and gene‒environment interactions on AMI risk in further studies.

Several studies have analyzed the effect of P2Y12 polymorphisms on platelet aggregation with inconsistent results. Prior studies on European and Egyptian populations have shown that the P2Y12 i744T > C polymorphism is associated with enhanced platelet aggregation in response to ADP [5, 8]. In a smaller study of 26 AMI patients with a 300 mg dose of clopidogrel from India [21], patients with the P2Y12 H2 haplotype demonstrated a trend toward an impaired response of clopidogrel to inhibit platelet aggregation. Similarly, several studies performed on different populations from Germany [28], Morocco [29], and the Guangzhou Province of China [30] suggested that the P2Y12 H2 haplotype was associated with altered platelet reactivity, thereby resulting in clopidogrel resistance (CR). However, several studies in other ethnicities, such as studies from North India [22], Mexico [19], and Iran [31], have not found any association between the P2Y12 H2 haplotype and CR. The contradictory results in different populations might be partly attributable to racial differences, selection criteria in the study population and methods used to assess platelet aggression. In the present study, we observed that carriers of the P2Y12 H2 haplotype displayed higher ADP-induced MAR than noncarriers among healthy volunteers in Quanzhou, but their association was not confirmed in AMI patients. However, we did not detect the baseline platelet aggregation rate and failed to assess the variability of platelet aggregation in patients before and after AMI development. Additionally, inter individual variations in the platelet aggregation rate may alter the results, and this is one of the limitations of our study. Thus, the effect of the P2Y12 H2 haplotype on platelet aggression needs to be further investigated.

The frequency of the 34 T allele was 20.6%, which is consistent (*P* > 0.05) with the findings in a Korean population (26.6%) (Table 6) [7]. Two publications reported that the P2Y12 c.34C > T polymorphism was not associated with platelet aggregation in response to ADP in northern European and Korean populations [5, 7]. However, several studies have reported that the P2Y12 c.34C > T polymorphism was associated with a high risk of clopidogrel resistance in Turkish and Han Chinese populations [32, 33], but this significant association was not found in a Caucasian population [34]. Our results suggest that P2Y12 c.34C > T polymorphisms do not correlate with the risk of AMI and ADP-induced platelet aggregation.

This study has some limitations. The major limitation is the relatively low number of both AMI patients and control subjects participating in our study. Additionally, a set of gene variations was found to be associated with AMI risk, while our study only assessed the influence of the P2Y1 and P2Y12 genes. Therefore, investigations into more selective genetic variations of the P2Y12 H2 haplotype in large sample sizes must be performed to elucidate the full extent of gene‒gene interaction effects on AMI pathogenesis. In addition, further studies should assess the platelet aggregation rate before and after AMI development to calculate the variability of platelet aggregation and investigate its correlation with P2Y1 and P2Y1 polymorphisms.

## Conclusion

In this study, we found that the P2Y12 H2 haplotype was associated with a higher risk of AMI. The P2Y12 H2 haplotype was associated with increased ADP-induced platelet aggression in healthy adults, but this association was not confirmed in AMI patients. Thus, the association between the P2Y12 H2 haplotype and platelet aggregation requires further investigation. Additionally, neither the P2Y1 c.1622A > G nor P2Y12 c.34C > T polymorphism was found to be associated with AMI susceptibility and MAR in response to ADP.

## Data Availability

The datasets used and/or analyzed during the current study are available from the corresponding author upon reasonable request.

## Abbreviations

AMI: Acute myocardial infarction
MAR: Maximal platelet aggregation rate
ADP: Adenosine diphosphate
CVDs: Cardiovascular diseases
CAD: Coronary artery disease
IS: Ischemic stroke
BMI: Body mass index
TG: Triglyceride
HDL-C: High-density lipoprotein cholesterol
LDL-C: Low-density lipoprotein cholesterol
TC: Total cholesterol
